# The Use of Phylogeny to Interpret Cross-Cultural Patterns in Plant Use and Guide Medicinal Plant Discovery: An Example from *Pterocarpus* (Leguminosae)

**DOI:** 10.1371/journal.pone.0022275

**Published:** 2011-07-18

**Authors:** C. Haris Saslis-Lagoudakis, Bente B. Klitgaard, Félix Forest, Louise Francis, Vincent Savolainen, Elizabeth M. Williamson, Julie A. Hawkins

**Affiliations:** 1 School of Biological Sciences, University of Reading, Reading, United Kingdom; 2 Division of Biology, Silwood Park Campus, Imperial College London, Ascot, United Kingdom; 3 Herbarium, Library, Art and Archives, Royal Botanic Gardens, Kew, Richmond, United Kingdom; 4 Jodrell Laboratory, Royal Botanic Gardens, Kew, Richmond, United Kingdom; 5 School of Pharmacy, University of Reading, Reading, United Kingdom; East Carolina University, United States of America

## Abstract

**Background:**

The study of traditional knowledge of medicinal plants has led to discoveries that have helped combat diseases and improve healthcare. However, the development of quantitative measures that can assist our quest for new medicinal plants has not greatly advanced in recent years. Phylogenetic tools have entered many scientific fields in the last two decades to provide explanatory power, but have been overlooked in ethnomedicinal studies. Several studies show that medicinal properties are not randomly distributed in plant phylogenies, suggesting that phylogeny shapes ethnobotanical use. Nevertheless, empirical studies that explicitly combine ethnobotanical and phylogenetic information are scarce.

**Methodology/Principal Findings:**

In this study, we borrowed tools from community ecology phylogenetics to quantify significance of phylogenetic signal in medicinal properties in plants and identify nodes on phylogenies with high bioscreening potential. To do this, we produced an ethnomedicinal review from extensive literature research and a multi-locus phylogenetic hypothesis for the pantropical genus *Pterocarpus* (Leguminosae: Papilionoideae). We demonstrate that species used to treat a certain conditions, such as malaria, are significantly phylogenetically clumped and we highlight nodes in the phylogeny that are significantly overabundant in species used to treat certain conditions. These cross-cultural patterns in ethnomedicinal usage in *Pterocarpus* are interpreted in the light of phylogenetic relationships.

**Conclusions/Significance:**

This study provides techniques that enable the application of phylogenies in bioscreening, but also sheds light on the processes that shape cross-cultural ethnomedicinal patterns. This community phylogenetic approach demonstrates that similar ethnobotanical uses can arise in parallel in different areas where related plants are available. With a vast amount of ethnomedicinal and phylogenetic information available, we predict that this field, after further refinement of the techniques, will expand into similar research areas, such as pest management or the search for bioactive plant-based compounds.

## Introduction

Thousands of plant species are used in traditional medicine around the globe, with almost one in four species on the planet used in traditional medicine in some culture [1]. For decades researchers have worked towards compiling a comprehensive list of medicinal plant species from different regions around the world. The documentation of such knowledge is crucial not only in order to preserve it, but also to understand patterns that shape this knowledge and to direct studies that can lead to the discovery of new medicinal plants. Indeed, in the last decades, the field of bioscreening has been guided by ethnomedicine, the study of traditional medicine, leading to the discovery of several plant-derived pharmaceuticals [2], [3], [4].

Medicinal properties are not randomly distributed in plants. Instead, some plant groups are represented by more medicinal plants than others [5], [6], [7], [8], [9]. Some of these studies suggested than when looking for new medicinal plants, one should sample from the “hot” groups, as they are more likely to deliver [7], [9]. Although this suggests that there is a phylogenetic pattern in medicinal properties, these studies were not explicitly phylogenetic. Phylogenetic conservatism [10], [11] in medicinal properties has been proposed [12], [13]. Lukhoba *et al.* [14] showed that for the genus *Plectranthus* (Lamiaceae), with 62 of the 300 species used in some sort of ethnomedicinal preparation, most medicinal species were found within the same large phylogenetic clade, suggesting there is a phylogenetic pattern in medicinal properties within the genus. Although this was not quantified, a later study by Forest *et al.* [15] used a more quantitative approach to show that in the Cape flora of South Africa, ethnomedicinal plants were significantly clumped on the phylogeny. A similar situation is observed in *Narcissus* species with medicinal properties [13]. The reason for this non-random phylogenetic distribution in medicinal properties might be that closely related plant species share biochemistry [16] and therefore, close relatives are likely to share medicinal properties. The presumption of shared chemistry in close relatives gave rise to the field of chemosystematics [17], [18], [19], [20], [21]. Nowadays taxonomies are no longer proposed based on chemical affinities; instead, phylogeny provides a framework to understand the distribution of chemistry. Combined phylogenetic and phytochemical studies have shown that there is strong phylogenetic signal in the distribution of chemical constituents in plants [22], [23], [24] that can be applied in the research for novel natural products [13], [25], [26], [27]. However, chemical data are unavailable for the majority of species and can be costly to generate. With less than a quarter of plant species screened for bioactivity [28], explicit tools are needed that can predict the phylogenetic position of species with high potential. The emerging field, which we refer to here as “phylogenetic ethnobotany”, still lacks quantitative metrics.

Biological phylogenies have proved to be extremely versatile and valuable tools that have been applied in various fields, in order to recover a variety of patterns, including biogeographical [29], [30], ecological [31], [32], [33], developmental [34], chemical [22], [23] and epidemiological [35]. With the exception of consideration of phylogenetic patterns in biodiversity conservation [15], [36], [37] and comparative sequence analyses to identify organisms (DNA barcoding) [38], [39], [40], [41], the potential of phylogenies to more applied fields has been overlooked. Aside from the field of bioscreening, phylogenetic patterns in medicinal plant use can enrich our understanding of traditional ethnobotanical knowledge. The finding that some plant lineages are more heavily used than others [5], [6], [7], [8], [9] and the fact that there is a degree of agreement in those lineages between disparate cultures [9], [42], [43] implies that phylogenetic relationships underlie people's selection of medicinal plants in traditional medicine and in a fashion that overcomes cultural differences. With the exception of some unpublished studies presented at ethnobotanical conferences [44], [45], [46], such findings have not been investigated in an explicitly phylogenetic framework. By superposing medicinal properties on lineages with wide distributions, one can observe cross-cultural phylogenetic patterns in ethnobotany, such as the agreement in usage of closely related lineages in distant cultures [44].

**Figure 1:**
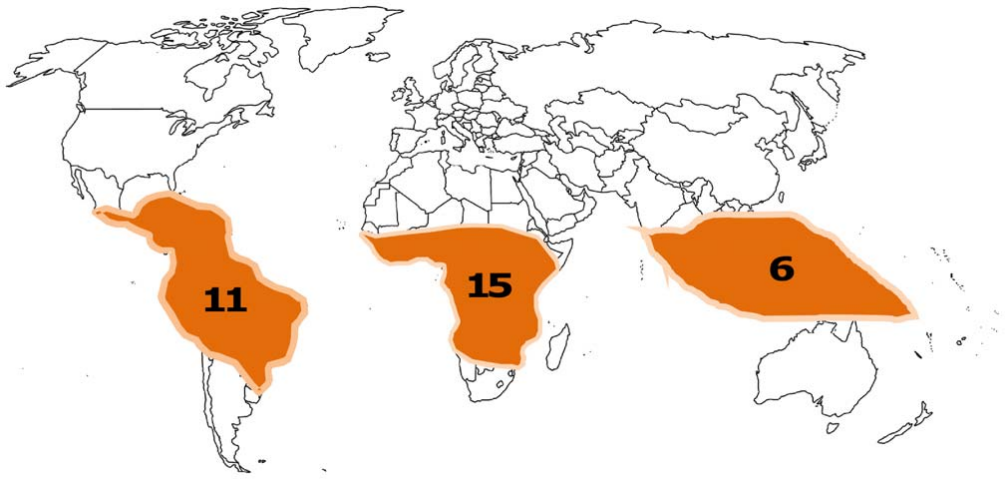
The pantropical distribution of *Pterocarpus*. Numbers indicate the numbers of taxa in different geographic regions; Neotropics, Tropical Africa, Indomalaya (Indian Subcontinent and Malay Peninsula/Archipelago).

*Pterocarpus* is a pantropical genus of dalbergioid legumes. It has been the subject of several regional taxonomic treatments [47], [48], [49], [50], [51] and one monographic study by Rojo [52]. In that study, Rojo recognised 20 species (23 taxa), but Lewis [53] estimated this number as 25–30 species, not supporting Rojo's synonymisation of several taxa under the American species *P. rohrii.* The most recent estimate is that of Klitgaard and Lavin [54], where the number of species was estimated as 35–40. The main centre of diversity of *Pterocarpus* is tropical Africa followed by the Neotropics and Indomalaya [52], as shown in Figure 1. Several *Pterocarpus* species are exploited throughout their range as timber as well as in traditional medicine. As Klitgaard and Lavin [54] state, the Indomalayan *narra* (*P. indicus*) is possibly one of the most important timber legumes globally, and several African species are very important timber trees known as *paduak*. The genus is used medicinally across its range for a variety of conditions. *Pterocarpus* species have received a lot of attention in recent years in experimental studies that have provided evidence for their bioactivity. Partly due to their extensive use, three species (*P. indicus, P. santalinus, P. marsupium*) are listed under the IUCN Red list of threatened species [55] and *P. santalinus* is also included in CITES Annex II. Because of the wide range of documented ethnomedicinal uses for *Pterocarpu*s species, the evidence of bioactivity for some of them, the critical status for some species heavily affected by usage and the distribution of the genus across three regions (Neotropics, tropical Africa and Indomalaya), it is an ideal model group to develop approaches to study phylogenetic patterns in medicinal properties.

### Objectives

The objectives of the present study are to: i) compile information from ethnobotanical sources to produce an ethnomedicinal review of *Pterocarpus* from the literature across its geographic range, ii) provide a phylogenetic hypothesis for the genus based on DNA sequence data, iii) develop methods that allow more explicit use of molecular phylogenetics in bioscreening, iv) highlight taxa that could have medicinal properties and have been overlooked, based on evidence from traditional medicine and the phylogeny and v) explore cross-cultural ethnomedicinal patterns across the range of the genus in light of phylogenetic relationships.

## Materials and Methods

### Ethnomedicinal information

Information on the medicinal uses of *Pterocarpus* species was compiled from extensive literature research from 125 sources, including published articles, online databases and local compendia of traditional medicine. All literature sources are given in Table S1. We collected information on the medicinal applications of *Pterocarpus* species in traditional medicine throughout the range of the genus, as well as pharmacological data from experimental studies. These applications were subsequently organised in 13 categories of use following [56]: Circulatory/Blood, Gastro-intestinal, Genito-urinary/Fertility, Infections/Fever, Inflammation, Musculo-Skeletal, Nervous, Pain, Poisons treatment, Respiratory, Sensory, Skin and Unspecific.

### Taxon sampling

Rojo [52] recognised 23 taxa in 20 species, but Lewis [53] estimated this number to be 25–30 species, not supporting Rojo's synonymisation of several taxa under the American species *P. rohrii*. Specifically, he recognised *P. ternatus*, *P. villosus* and *P. zehntneri* as separate species from *P. rohrii* and we follow this taxonomy here. We included all taxa recognised by Rojo [52] (with the exception of the infraspecific taxon *P. indicus* forma *echinatus* due to material unavailability), accepting the infraspecific divisions of *P. rotundifolius* [57], [58] and of *P. mildbraedii* [59], and adding two neotropical taxa described after Rojo's monographic work, namely *P. michelianus* [60] and *P. monophyllus* [61]. This brings the total taxa recognised in this study to 30 in 25 species. Finally, we sampled several of the species that have been placed in synonymy under the species complexes *P. rohrii* and *P. tinctorius*. All *Pterocarpus* specimens included in the analyses are shown in Table S2. Outgroups were selected from previous phylogenetic analyses of dalbergioid legumes [62], [63], [64], [65]. We sampled genera closely related to *Pterocarpus*: *Centrolobium*, *Grazielodendron*, *Inocarpus*, *Maraniona*, *Ramorinoa*, *Tipuana*. *Platymisicum* was used as external outgroup taxon for the clade comprising these genera and *Pterocarpus* and defined as such in all analyses. Outgroup accessions are shown in Table S3.

### Selection of DNA markers

We selected DNA markers based on amplification efficiency and variability. We used the plastid regions *rbcL* and *matK* that have shown great amplification efficiency across the angiosperms and the legume family [66], [67], [68] and have been successfully amplified and served as barcodes for two species of *Pterocarpus* in the literature [39]. Additionally, we selected the *ndhF-rpL32* intergenic spacer, a plastid marker shown to be potentially one of the most variable within the majority of angiosperm groups in a scan of the plastid genome [69]. Finally, we amplified nrITS2 and the *trnL-F* intergenic spacer, since these regions have provided phylogenetic resolution for closely related genera in previous studies [63], [64], [65], [70].

### DNA extraction and sequencing

Total DNA was extracted from 0.2 to 0.3 g of leaf and/or flower tissue from herbarium or silica gel dried material using a modification [71] of the Doyle and Doyle method [72]. DNA was purified using QIAquick columns (Qiagen, Crawley, West Sussex, UK) following the manufacturer's protocol.

The internal transcribed spacer 2 (ITS2), including parts of the 5.8S ribosomal RNA gene and the 26S ribosomal RNA gene, was amplified using primers ITS3 and ITS26E [73].The PCR protocol included a 2 min initial denaturation at 96°C and 32 cycles of 1 min denaturation (96°C), 1 min annealing (48°C), 50 s elongation (72°C), with a final elongation of 7 min at 72°C. The *trnL*-*F* intergenic spacer was amplified with primers “e” and “f” [74]. The PCR protocol included a 4 min initial denaturation (96°C) and 32 cycles of 1 min denaturation (96°C), 1 min annealing (54°C), 1 min elongation (72°C) and final elongation of 7 min at 72°C. The barcoding fragment of *matK* was amplified with primers X and 3.2 [75]. The PCR protocol included a 1 min initial denaturation (96°C) and 38 cycles of 30 s denaturation (96°C), 40 s annealing (46°C), 1 min elongation (72°C), with a final elongation of 7 min at 72°C. The first half of *rbcL* was amplified with primers rbcL1F and rbcL724R [76], following a protocol of 4 min initial denaturation (96°C), and 33 cycles of 1 min denaturation (96°C), 1 min annealing (50°C) and 1 min 20s elongation (72°C), with a final elongation of 7 min at 72°C. Finally, the *ndhF-rpL32* intergenic spacer was amplified with primers *ndhF* and *rpL32*-R [69]. Due to amplification of non-target product, we modified the PCR conditions given by [69] as follows: one cycle of denaturation (96°C) for 2 min, 30 cycles of 95°C for 40 s, 52°C for 1 min and 65°C for 3 min 20 s with ramp of 0.3/s to 65°C and a final elongation cycle of 65°C for 5 min. All amplifications were performed in 30-µL volume reactions with BioMix (Bioline Ltd. London, UK).

PCR purification and DNA sequencing of both strands were performed by Macrogen Inc. (Seoul, Korea). Complementary strands were assembled and edited with EditSeq (DNASTAR, Madison, WI). Alignments for *rbcL* and *matK* sequences were performed manually in BioEdit v. 7.0. ITS2, and the *trnL-F* and *ndhF-rpL32* intergenic spacer sequences were aligned using CLUSTAL W [77], and adjustments were made manually in BioEdit v. 7.0, following the guidelines of Kelchner [78]. All newly generated sequences have been submitted to GenBank (see Tables S2 and S3) and the data matrix and phylogenetic tree generated here are available on TreeBase (www.treebase.org) under the accession number 11586.

### Phylogenetic analyses and manipulations

Sequence data were analysed under the Maximum Likelihood (ML) criterion, with RAxML [79] using the partitioned model option with the GTR+Γ model and running 1000 bootstrap replicates [80].

We borrowed two metrics from community ecology phylogenetics in order to assess and detect phylogenetic signal in medicinal properties. The first was the “comstruct” option in Phylocom 4.1 [81]. This metric assesses the significance of phylogenetic signal for a community of taxa, which is the subset of a phylogeny. In other words, it calculates how significantly a group of species are clumped on the phylogeny. To do this, the mean phylogenetic distance (MPD) and mean nearest phylogenetic taxon distance (MNTD) for each sample (group of species on the phylogeny) is calculated and they are compared to MPD/MNTD values for randomly generated samples to provide p values for the significance of phylogenetic signal for the given sample (p values are calculated based on the frequency of random samples that were more clumped on the phylogeny than the real sample). For this study, we compiled “communities” of taxa that are used for one of the categories of use. This means that instead of grouping taxa based on which ecological zone or geographical area they are found, we grouped taxa that have similar uses in medicine together under one “community”. This way, we are able to assess the phylogenetic signal of each category of use on the phylogeny of *Pterocarpus* and answer the question: Are taxa used for a certain category more significantly related than expected by chance alone?

The second metric used was the command “nodesig” in Phylocom v 4.1 [81]. This option uses the same community sample as described above and tests each node of the phylogeny for overabundance of terminal taxa distal to it. Observed patterns for each sample are compared to those from random samples to provide significance for the observed overabundance. For a node that is identified through this approach, the descendants of this node are significantly more likely to belong to the “community” under consideration that expected by chance alone. As mentioned earlier, a “community” for this study represents the group of species used for a certain category of use. Hence, this technique identifies the exact position of phylogenetic clumping on the phylogeny, namely the “hot” nodes for a category of use. This can help us assess the predictive power of the phylogeny for the discovery of new medicinal species.

**Figure 2:**
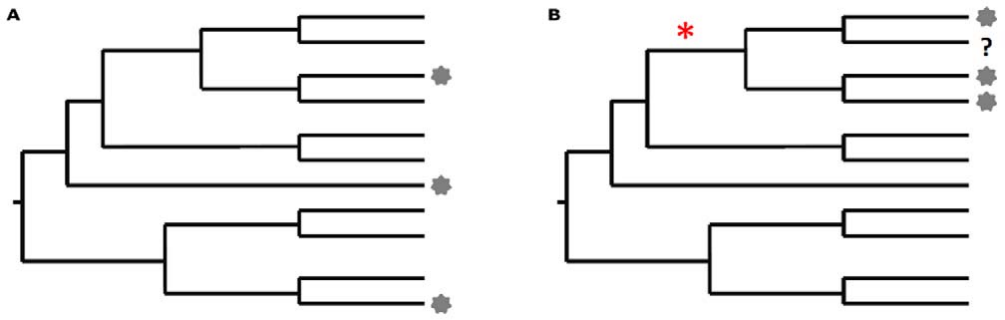
Two different scenarios for the distribution of medicinal uses on a hypothetical phylogeny. In both cases there are three medicinal taxa, designated at the tips of the tree. A: There is no significant phylogenetic signal as the taxa are overdispersed. B: The phylogenetic signal is strong as three of the four closely related species are used and the node indicated with “*” shows significant overabundance in medicinal species. In the first case phylogeny cannot act as a guide for discovery of medicinal species. In the second case the species marked with “?” potentially shares medicinal properties with its close relatives.

The rationale behind using these metric is as follows: If a certain category of use shows strong phylogenetic signal, then closely related species demonstrate similar uses. With the first metric, we can asses which categories of use demonstrate strong phylogenetic signal. For these categories of use, we can subsequently identify which nodes on the phylogeny have more medicinal taxa than expected by chance, using the second tool. Taxa descending from these nodes are the ones that show significant “overabundance” in medicinal properties. Therefore, they deserve further investigation, including those species that are not reported in traditional medicine, as they are likely to share these properties with their relatives, as shown in Figure 2. The matrix showing the samples used for all Phylocom analyses is given in Table S4.

Analyses using these two approaches were carried out for each of the 13 categories of use mentioned above. Additionally, we performed the same analyses for three diseases of particular interest for which there is experimental evidence of bioactivity of *Pterocarpus* species: diabetes, malaria and cancer [82], [83], [84], [85], [86], [87], [88], [89], [90], [91], [92], [93]. This also allowed a test of our methods at different levels of ethnomedicinal specificity (condition versus group of conditions).

## Results

### Ethnomedicinal review

**Figure 3:**
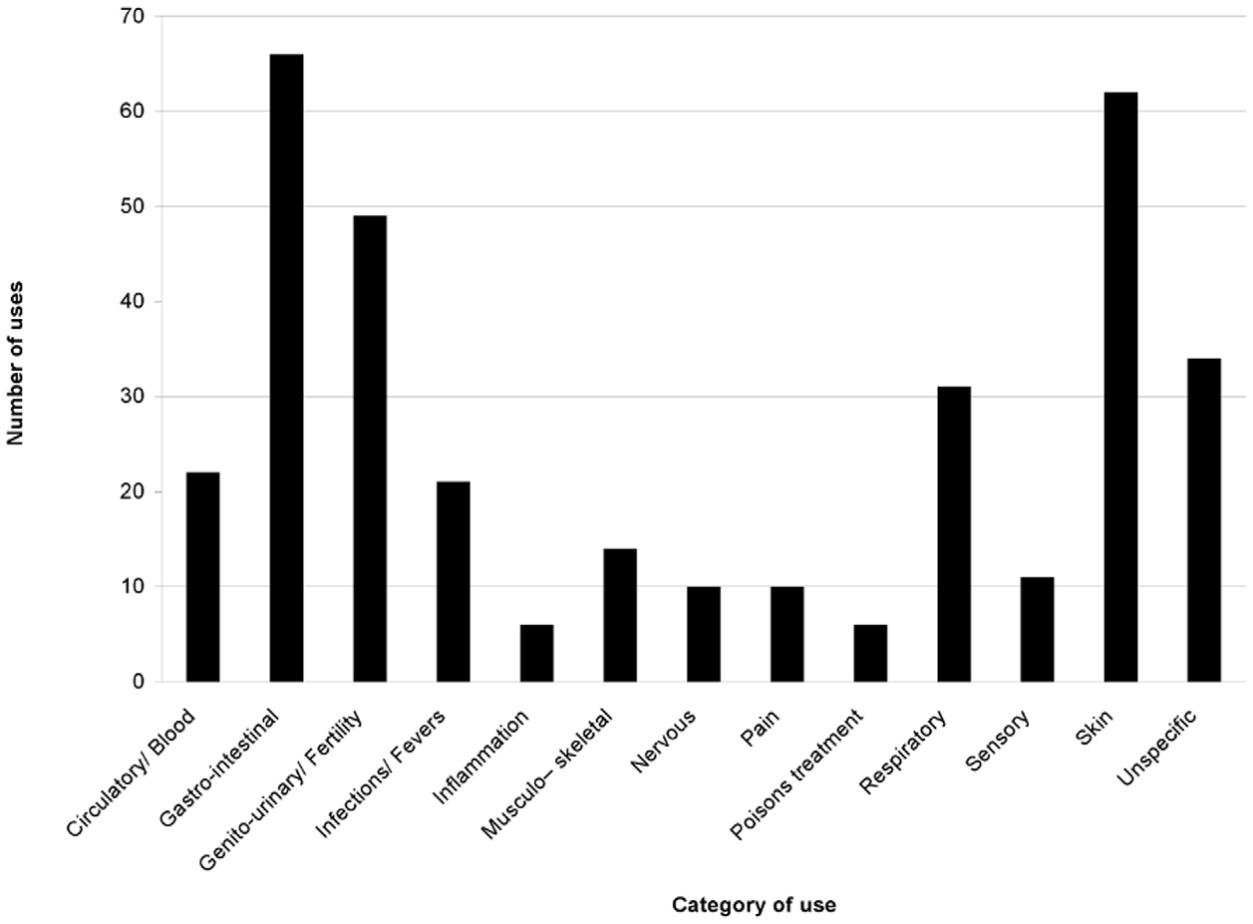
Number of uses per category of use for *Pterocarpus* species.

**Figure 4:**
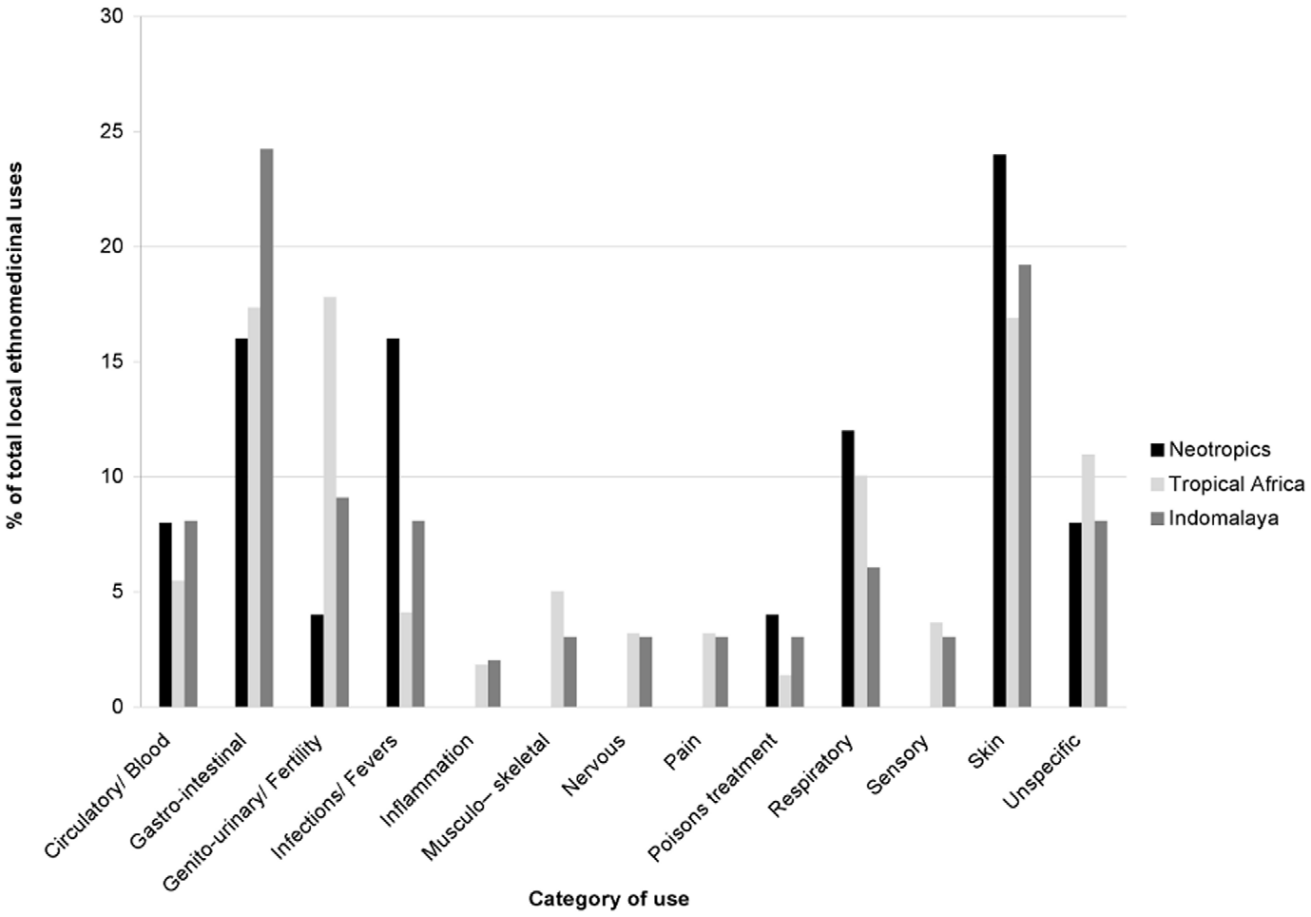
Relative usage per category of use for *Pterocarpus* in the Neotropics, Tropical Africa and Indomalaya.

Medicinal properties found in the literature for *Pterocarpus* species are shown in Table S1. Nineteen taxa are found with some medicinal applications and the species with the greatest numbers of reported uses are the African *P. erinaceus* (65), *P. angolensis* (56), *P. soyauxii* (37) and the Indomalayan *P. santalinus* (43) and *P. indicus* (32). As shown in Figure 3, *Pterocarpus* species are mainly used for Gastro-intestinal and Skin problems but they also have wide applications for Genito-urinary/Fertility and Respiratory conditions. Anti-inflammatory and poison remedies are the least common. The usage patterns of *Pterocarpus* species are fairly similar across all three regions (Neotropics, Tropical Africa and Indomalaya) of the pantropical range of the genus. For example, Gastro-intestinal and Skin remedies are consistently the most common, while Inflammation Nervous and Pain treatments are the least common in all three regions (Figure 4). One of the most profound differences between the three regions is the heavy use of neotropical taxa to treat Infections/Fever and their low contribution to Genito-urinary treatments, one of the most common uses in tropical Africa and Indomalaya.

### Phylogenetic analyses

**Figure 5:**
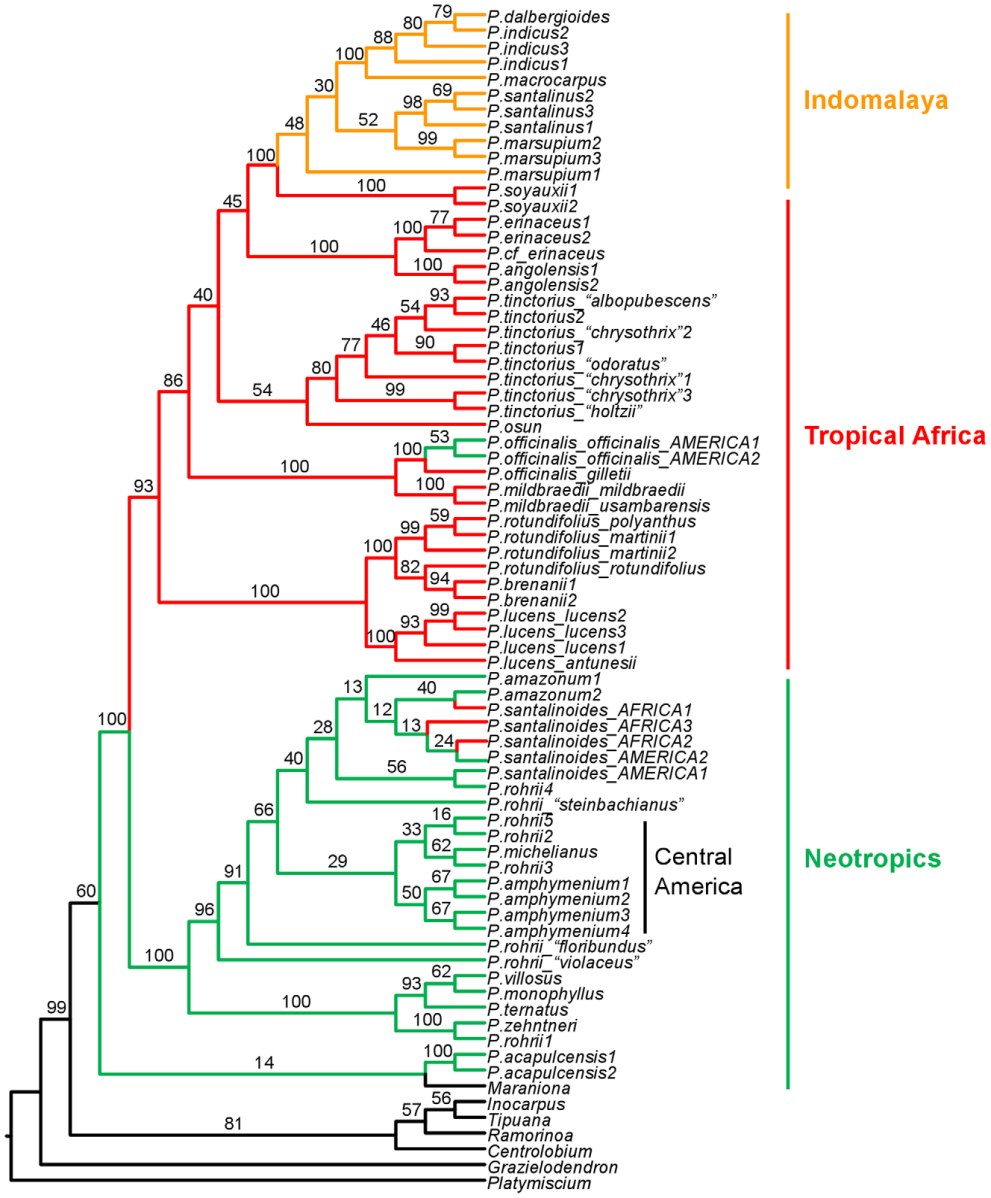
ML phylogenetic tree of *Pterocarpus* species and allies. The tree was reconstructed with RAxML and using all DNA markers (nrITS2, *rbcL*, *matK*, *trnL* and *ndhF-rpL32*). Numbers above branches show bootstrap percentages (BP). Distributions of the main clades are on the right.

The matrix included 75 taxa, 68 of which were *Pterocarpus* taxa and seven were closely related genera. The total length of the aligned matrix was 3,592 bp. Phylogenetic reconstruction analysis with RAxML produced the phylogenetic tree shown in Figure 5. *Pterocarpus acapulcensis*, weakly resolved with the outgroup monospecific genus *Maraniona*, is placed in a sister relationship with the rest of the genus. The rest of the genus is divided into two large clades, one comprising the species complex *P. rohrii* and the rest of the neotropical taxa (BP 100) and the other including all African and Indomalayan taxa (BP 93), the latter nested within the African grade (Figure 5). Several species are not recovered as monophyletic, although most without strong support, except for *P. rohrii*.

### Phylogenetic manipulations

The assessment of phylogenetic signal, recovered with the “comstruct” tool, is shown in Table **1**. Medicinal usage overall was not phylogenetically clumped, meaning that *Pterocarpus* species used medicinally are not found in a certain lineage, but are distributed all over the phylogeny of the genus. However, when the usage was organised in categories we observed some cases of strong phylogenetic signal. The only category of use that showed significant phylogenetic clumping with the MPD was Musculo-skeletal. In contrast, there were six uses (Inflammation, Musculo-Skeletal, Pain, Sensory, Skin and Malaria) that demonstrated significant phylogenetic signal with the MNTD (Table **1**).

**Table 1:** Significance (p values) of phylogenetic clumping of medicinal usage of *Pterocarpus* species, assessed with the “comstruct” option in Phylocom v4.1.

Category of use	p value (MPD)	p value (MNTD)
Medicinal uses overall	>0.05	>0.05
Circulatory/Blood	>0.05	>0.05
Gastro-intestinal	>0.05	>0.05
Genito-urinary/Fertility	>0.05	>0.05
Infections/Fevers	>0.05	>0.05
Inflammation	>0.05	**<0.05**
Musculo-skeletal	**<0.05**	**<0.01**
Nervous	>0.05	>0.05
Pain	>0.05	**<0.05**
Poisons treatment	>0.05	>0.05
Respiratory	>0.05	>0.05
Sensory	>0.05	**<0.05**
Skin	>0.05	**<0.05**
Unspecific	>0.05	>0.05
Diabetes	>0.05	>0.05
Malaria	>0.05	**<<0.01**
Cancer	>0.05	>0.05

Numbers in bold indicate cases where significant phylogenetic signal was recovered.

The nodes that demonstrated significant overabundance in medicinal species with the “nodesig” command in Phylocom v4.1 for Inflammation, Musculo-Skeletal, Pain, Sensory, Skin and Malaria uses are shown in Table 2. With few exceptions, most of the nodes are located in the clade comprising the African and the Indomalayan species and there is great overlap in the “overabundant” nodes across the uses.

**Table 2:** Nodes recovered as significantly overabundant in medicinal species in the *Pterocarpus* phylogeny, as assessed with the “nodesig” option in Phylocom v4.1.

Category of use	node defined as the MRCA* of
Inflammation	*P.lucens_antunesii-P.dalbergioides*
Inflammation	*P.mildbraedii_usambarensis-P.dalbergioides*
Inflammation	*P.osun-P.dalbergioides*
Inflammation	*P.angolensis2-P.dalbergioides*
Inflammation	*P.angolensis2-P.erinaceus1*
Inflammation	*P.soyauxii2-P.dalbergioides*
Inflammation	*P.marsupium3-P.santalinus2*
Musculo-skeletal	*P.lucens_antunesii-P.dalbergioides*
Musculo-skeletal	*P.mildbraedii_usambarensis-P.dalbergioides*
Musculo-skeletal	*P.osun-P.dalbergioides*
Musculo-skeletal	*P.angolensis2-P.dalbergioides*
Musculo-skeletal	*P.angolensis2-P.erinaceus1*
Musculo-skeletal	*P.soyauxii2-P.dalbergioides*
Musculo-skeletal	*P.marsupium3-P.santalinus2*
Pain	*P.lucens_antunesii-P.dalbergioides*
Pain	*P.mildbraedii_usambarensis-P.dalbergioides*
Pain	*P.osun-P.dalbergioides*
Pain	*P.angolensis2-P.dalbergioides*
Pain	*P.angolensis2-P.erinaceus1*
Pain	*P.soyauxii2-P.dalbergioides*
Pain	*P.marsupium1-P.dalbergioides*
Pain	*P.marsupium3-P.santalinus2*
Sensory	*P.lucens_antunesii-P.dalbergioides*
Sensory	*P.mildbraedii_usambarensis-P.dalbergioides*
Sensory	*P.osun-P.dalbergioides*
Sensory	*P.osun-P.tinctorius_"albopubescens"*
Sensory	*P.tinctcorius_"holtzii"-P.tinctorius_"albopubescens"*
Sensory	*P.angolensis2-P.dalbergioides*
Sensory	*P.angolensis2-P.erinaceus1*
Sensory	*P.marsupium1-P.dalbergioides*
Sensory	*P.marsupium3-P.santalinus2*
Skin	*P.mildbraedii_usambarensis-P.dalbergioides*
Skin	*P.osun-P.dalbergioides*
Skin	*P.osun-P.tinctorius_"albopubescens"*
Skin	*P.tinctorius_"holtzii"-P.tinctorius_"albopubescens"*
Skin	*P.angolensis2-P.dalbergioides*
Malaria	*P.osun-P.dalbergioides*
Malaria	*P.angolensis2-P.dalbergioides*
Malaria	*P.angolensis2-P.erinaceus1*
Malaria	*P.marsupium3-P.dalbergioides*
Malaria	*P.macrocarpus-P.dalbergioides*
Malaria	*P.indicus1-P.dalbergioides*
Malaria	*P.rohrii_"steinbachianus"-P.amazonum1*
Malaria	*P.rohrii4-P.amazonum2*

*Most Recent Common Ancestor.

## Discussion

In this study we produced an ethnomedicinal review for the genus *Pterocarpus* (Table S1) and reconstructed the relationships between all *Pterocarpus* species, presenting a well supported molecular multi-locus phylogeny for the genus (Figure 5). Using these tools, we assess the proposed application of phylogenetics to bioscreening and ethnobotany [12], [13], [14], [15] and devise meaningful tools that can predict the phylogenetic position of species with high medicinal potential. Some of the phylogenetic relationships recovered here have been hypothesised based on morphological affinities, adding support to our results. These include the proximity between *Pterocarpus mildbraedii* and *P. officinalis, P. amazonum* and *P. santalinoides*, *P. brenanii* and *P. rotundifolius* [52], *P*. *monopyllus* and *P. ternatus* [61] and between the five Indomalayan species [52]. As mentioned above it has, however, long been suspected that several *Pterocarpus* species are paraphyletic - e.g. the Neotropical species *Pterocarpus rohrii* of which the samples included in this study are found in scattered position across the Neotropical clade. Recognising the necessity for well-circumscribed taxonomic entities in useful plants groups, one of us (BBK) is currently undertaking a taxonomic revision of *Pterocarpus*.

In terms of ethnomedicinal uses, our results from an extensive literature review indicate that *Pterocarpus* is a very valuable genus in traditional medicine, as almost two thirds of the taxa are used throughout the range of the genus and for multiple uses. Although we found usage under several of the categories suggested by [56], *Pterocarpus* species are mainly used for Gastro-intestinal and Skin afflictions but they also have wide applications for Genito-urinary/fertility and respiratory conditions, as shown in Figure 3. The well supported phylogeny of all species in *Pterocarpus*, along with its richness in medicinal uses, provided a suitable model to test phylogenetic patterns in medicinal properties and allowed us to perform explicit phylogenetic tests.

We detected strong phylogenetic signal in medicinal usage in several cases, indicating that medicinal properties in the genus are not distributed evenly across the phylogeny, but are rather clumped, as was suggested in previous studies of other groups at different hierarchical levels (genus [13], [14] and flora [15]). More specifically, usage for inflammations, musculo-skeletal afflictions, pain, sensory and skin problems, as well as malaria, demonstrated significant clumping on the phylogeny (Table **1**). Although most of these categories were the ones with few uses, they also include uses for skin problems, the second most commonly encountered category (Figure 3). As shown in Table **1**, phylogenetic signal was recovered mainly using the MNTD and not the MPD, where significant signal was found for one category of use only. These two values both measure phylogenetic clumping, however at different hierarchical levels. With the MPD measure, one can detect phylogenetic signal in deep nodes of the phylogeny, whereas with the MNTD clumping is measured towards the tips of the phylogenetic tree [94]. In advising bioscreening schemes, one would like to narrow down selection of putatively useful species to a small number. Therefore, indentifying clumping in deeper nodes of the phylogeny is probably not useful, as deep nodes define clades with numerous species, which means informed and well-defined decisions cannot be made for bioscreening. Thus, clumping toward the tips of the phylogeny (MNTD) is more relevant to bioscreening.

It has been proposed that cross-cultural agreement in plant usage implies bioactivity as independent discovery in disparate cultures should have an empirical basis [9], [95], [96], [97], [98]. Even without taking phylogenetic relationships into account, a degree of agreement among different ethnomedicinal systems is evident. Figure 4 shows that *Pterocarpus* species are used to treat similar conditions in the Neotropics, Tropical Africa and Indomalaya. Given the geographical distance of these three regions and the disparate cultures found there, it is very likely that this parallel usage is the product of independent discoveries, which demonstrates the efficiency of local cultures in identifying plants with relatively similar chemical profiles (the three biogeographical clades within *Pterocarpus*) to treat similar conditions. Undoubtedly, cultural exchange has taken place to a certain degree between these regions. For example, uses of *Ocimum* species have been recorded in Afro-Brazilian communities, attributed to traditional uses in Africa [99]. Although we acknowledge the possibility that common patterns might be due to cultural exchange, given the large geographic scale of this study, we believe such cases are the exception, rather than the rule. However, we recognise that common ethnobotanical trends, even when independent, might not be the result of underlying bioefficacy in every case. Plant use is often guided by a “doctrine of signatures”, the belief that a plant possess medicinal properties due the presence of physical attributes (colour, scent, shape) [100], [101]. The yellow flowers and red sap found in *Pterocarpus* species could be a reason of their applications in urinary and blood disorders. Nevertheless, despite all these possible alternative explanations as to how cross-cultural ethnobotanical patterns arise, we show that phylogenetic interpretation of such patterns allows us to address traditional questions in ethnobotany from novel perspectives.

The two amphiatlantic species (*P. officinalis* and *P. santalinoides*) provide an excellent system to study the use of the same species in notably different medicinal systems, in the light of phylogeny and biogeography. As Figure 5 shows, *P. officinalis* dispersed from West Africa to the Neotropics, as the neotropical subspecies (*P. officinalis* subsp. *officinalis*) is nested in an African clade, while *P. santalinoides* dispersed from the Neotropics to West Africa, as the African samples are nested in the neotropical clade. Interestingly, both taxa have more uses in the “new” regions than in their regions of origin and we attribute this pattern to phylogenetic structure. We recorded no uses for *P. officinalis* in Africa and six uses in the Neotropics. Similarly, we found one use for *P. santalinoides* in the Neotropics and 22 in Africa. These species, by having no close relatives in the new regions, contribute novel phylogenetic diversity, and hence possibly novel medicinal properties, to these areas. On the contrary, in the region of origin, close relatives with similar phytochemical profiles are available. For example, *P. santalinoides* is used for malaria in West Africa, but not in the Neotropics, where its close relatives *P. amazonum* and *P. rohrii* are used (Table S1). Similarly, *P. officinalis* is used in the Neotropics as an astringent, however that use is replaced in Africa, where it is very narrowly distributed, by *P. angolensis* and *P. erinaceus,* the latter being sympatric to *P. officinalis.* Moreover, we found common amphiatlantic use for *P. santalinoides* as a poison antidote. Such agreement in use has been found to be strongly linked to pharmacological activities at this taxonomic level [97].

**Figure 6:**
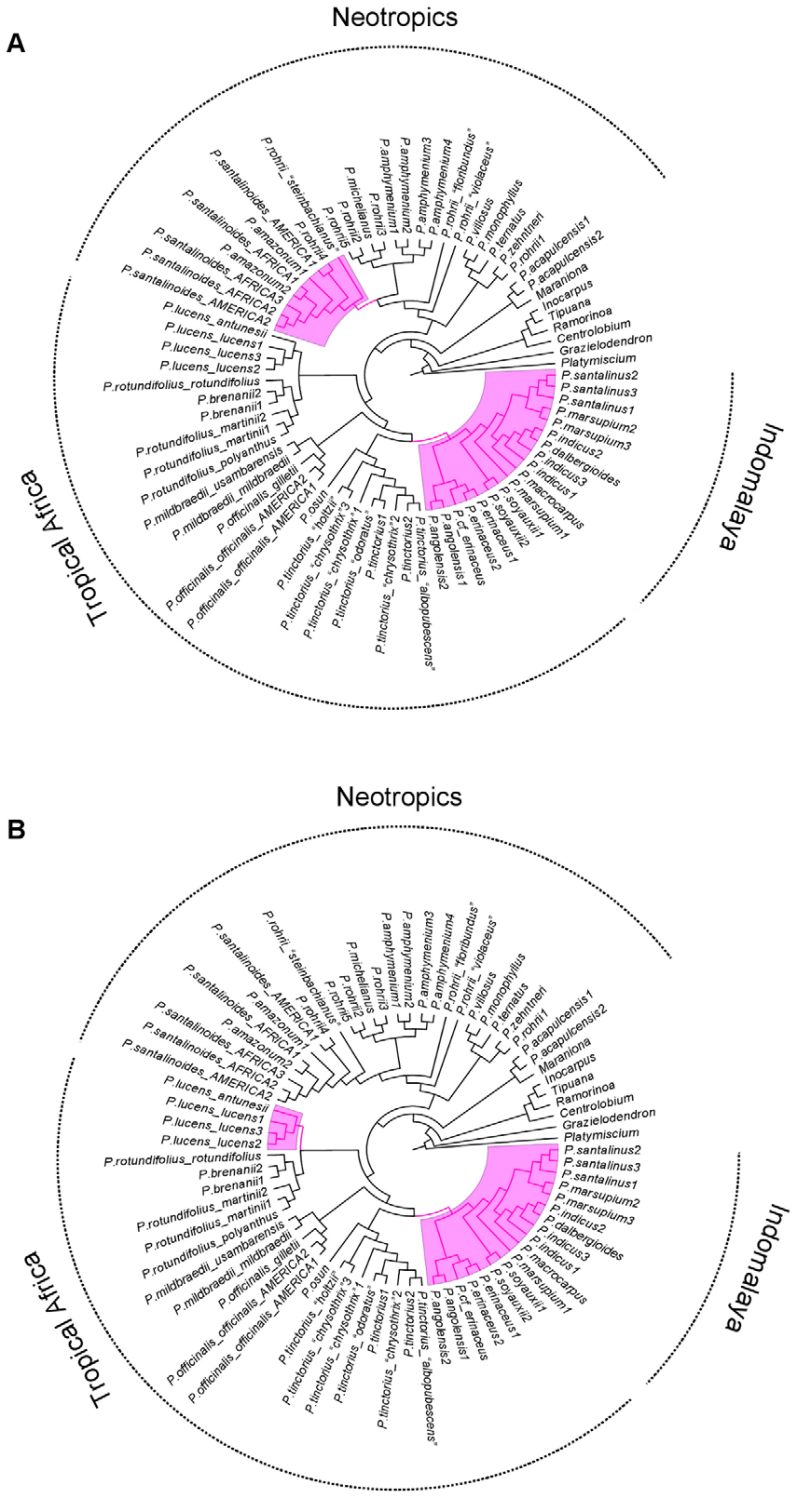
Phylogeny of *Pterocarpus* with clades that show significant overabundance in medicinal species highlighted. Results were recovered using the “nodesig” option in Phylocom v 4.1. A: species to treat malaria. B: species to treat musculo-skeletal conditions. Although some clades are used for a variety of conditions, different properties are found in different parts of the phylogeny.

Just as knowledge of phylogeny informs the interpretation of ethnobotanical use at the species level, confidence in inferences of bioactivity is increased when clades sharing specific ethnomedicinal uses are distributed across regions. For example, Figure 6 shows that the larger of the clades showing use in treating malaria and musculo-skeletal disorders is distributed in Tropical Africa and Indomalaya. As we discuss below, clades which encompass many species for a specific use can become targets for future screening. When these clades are distributed across regions, it seems more probable that selection for ethnomedicinal use reflects underlying activity, and not a preference within a culture for using species which might share particular attributes such as similar overall morphology, because of shared ancestry.

Regarding ethnopharmacology and bioscreening, there are three ways in which our results can be of use. First, as proposed in earlier investigations, close relatives of species with known bioactivity can be prioritised for screening for similar activity [12], [13]. For example, the species *P. santalinus* and *P. marsupium* are very well known species in traditional medicine, especially for their use to treat diabetes [102], [103], [104], [105]. Both species have been studied *in vitro* and have shown notable hypoglycaemic bioactivity [87], [88], [90], [91], [92], [93]. However, *P. santalinus* is listed as endangered and *P. marsupium* as vulnerable on the IUCN Red List [55] and the former is also included in CITES Annex II, therefore their use in medicine is not recommended as overharvesting could pose further threat to their survival. *Pterocarpus dalbergioides*, a stenoendemic of the Andaman Islands, has been shown to possess similar bioactivity [89], however its narrow range would not support sustainable harvesting either. Although the use for diabetes does not demonstrate significant phylogenetic structure on the phylogeny (Table **1**), these results suggest that hypoglycaemic bioactivity is shared by all species in the clade defined by the MRCA of *P.marsupium1-P.dalbergioides*, which includes *P. macrocarpus* and *P. indicus* that are widespread in Southeast Asia [52]. We propose that these widespread species be investigated for hypoglycaemic bioactivity to investigate whether they can substitute the use of the more endangered relatives. Should these species prove to share this bioactivity as we predict here, their application will not only provide new medicinal species, but will also assist the conservation of the more restricted and endangered species that are currently used.

Second, in the case of absence of pharmacological data, phylogenetic signal can provide indirect evidence of underlying bioactivity. If closely related species share similar ethnomedicinal properties (which can be interpreted as a case of phylogenetic conservatism [10], [11]), it is very likely that this reflects the underlying bioactivity of these species. For example, the clade comprising species from Africa and Indomalaya is the richest in medicinal properties. The species with the highest numbers of uses, namely *P. erinaceus* (65), *P. angolensis* (56), *P. santalinus* (43), *P. soyauxii* (37) and *P. indicus* (32) are all included in the clade defined by the MRCA of *P.angolensis2-P.dalbergioides* (Figure 5) that is often recovered among the nodes that show significant phylogenetic overabundance for different uses (Table 2). These species and their close relatives are therefore considered to be of high potential for bioprospecting. What is particularly interesting in this clade is that it is distributed in two large biogeographic regions, where very different human cultures are found and it is relatively safe to assume that any common ethnobotanical patterns observed in the two regions were discovered independently and are not due to shared cultural history. Therefore, not only does this clade demonstrate phylogenetic conservatism [10], [11] in medicinal usage, but it also demonstrates cross-cultural agreement in usage (Figure 4 and 6), which has been used as a criterion to imply bioactivity [9], [95], [106], [107]. These two criteria provide multiple lines of evidence pointing towards the bioactivity in this clade, especially for the conditions where significant clumping was observed (inflammations, musculo-skeletal afflictions, pain, sensory and skin problems, as well as malaria; Table **1**).

Third, a more sophisticated approach is to identify nodes on the phylogeny that have high potential for bioscreening. We demonstrated that with the tool “nodesig” in Phylocom the exact phylogenetic position of overabundance in medicinal properties can be recovered. For example, several *Pterocarpus* species are being used to treat malaria (Table S1) and our results show that the species used in such applications are significantly clumped on the phylogeny (Table 1), suggesting that phylogenetic proximity is a good proxy for antiplasmodial bioactivity. We can subsequently identify the nodes that are significantly overabundant in “antimalarial” species. These are given in Table 2 and also shown highlighted in Figure 6. As shown, there are two positions in the phylogeny that are overabundant in species with antimalarial activity and they cover all three regions of the range of the genus, again showing both phylogenetic conservatism and cross-cultural usage as evidence for bioactivity.

The first clade is a neotropical clade that includes *P. amazonum*, some *P. rohrii* samples and *P. santalinoides,* the last also found in West Africa. All three species are reported with demonstrable *in vitro* use against malaria [83], [84], [86]. The bioactivity for the amphiatlantic *P. santalinoides* was demonstrated for West African material [84], however as we show here, South American material is extremely likely to share these properties as it falls within this clade and we propose it be further investigated. *Pterocarpus rohrii* is an extremely variable and widespread species, found throughout South and Central America. The results from this study, which has sampled material across the species range, reveal the polyphyly of this species and show that phylogenetic units within the species show geographic structure (Figure 5) suggesting that its taxonomy should be revised. The samples in this “antimalarial” clade are from South America and bioactivity has been demonstrated for South American material only [83]. Based on our results, material of *P. rohrii* from this clade is more valuable as antimalarial, as the other lineages of *P. rohrii* are not recovered significantly overabundant in antimalarial use. Although it is not unlikely that this species possesses bioactivity throughout its range, but it is simply not used across its range due to differences in ethnomedicinal floras in different cultures, it is also possible that antimalarial activity is present in this clade only. Further research in this species on material from different localities is needed to establish whether antimalarial properties are present across its range. Nonetheless, the combination of traditional knowledge and phylogenetic information has already brought to light cryptic diversity demonstrating to be a valid approach to elucidating taxonomy [108] and we believe that such information could be incorporated in a taxonomic revision of *P. rohrii*, as it could clarify which taxonomic units are more valuable in ethnomedicine.

The second antimalarial clade includes all species defined by the MRCA of *P. osun* and *P. dalbergioides* (Table 2). Nevertheless, the only species in this clade that have reported antimalarial uses are *P. angolensis*, *P. erinaceus* (also *in vitro*), *P. indicus* and *P. macrocarpus*. This renders all other species in the clade, namely *P. dalbergioides*, *P. marsupium*, *P. osun*, *P. santalinus*, *P. soyauxii*, *P. tessmanii* and *P. tinctoriu*s very good candidates for antiplasmodial activity. Out of these, of particular interest are *P. soyauxii* and *P. tinctoriu*s, as they are widespread in Africa, material availability will be greater and no harvesting pressure will be posed to narrowly distributed or endangered species. The phylogenetic position of the former, which is closely related to *P. angolensis*, *P. erinaceus*, as well as to *P. indicus* and *P. macrocarpus* (Figure 5) makes it a better candidate. Furthermore, we predict that *P. angolensis*, already used traditionally as an antimalarial, will very likely share the *in vitro* activity of its sister species *P. erinaceus*.

### Conclusions

This, to the best of our knowledge, is the first multidisciplinary study that draws on four different sources (using taxonomic, phylogenetic, biogeographic and ethnobotanical information) to provide new perspectives on bioactivity in plants, based on the criteria of cross-cultural usage and phylogenetic conservatism across different biogeographic regions. Our study demonstrates that phylogeny and biogeography can be used as novel tools in ethnobotany to interpret processes that shape traditional usage and particularly cross-cultural patterns and our community phylogenetic approach demonstrates that similar ethnobotanical uses can arise in parallel in different areas when related plants are available there.The advent of molecular phylogenetics heralded a much deeper understanding of organismal relationships. Phylogenetic tools entered several disciplines to provide explanatory power and recover patterns previously undetected. Molecular data are becoming increasingly available in recent years, especially with the rapid development of next-generation sequencing techniques. At the same time, ethnomedicinal and ethnopharmacological information has also been accumulating over the last decades, providing invaluable insight into the use of nature by humans in traditional medicine. We demonstrated here that the combination of information from these fields using quantitative metrics is particularly meaningful and opens up new opportunities for further biological studies through its potential to direct bioscreening studies, but also enables insights into processes that shape ethnobotanical knowledge. With molecular and ethnomedicinal data publicly available and readily accessible, the potential for them to be combined and reanalysed reciprocally is immense.

These approaches could be developed even further than in this study. For example, ethnomedicinal metrics of confidence in plant use (relative cultural importance index [109], or informant consensus [98]) can be mapped on phylogeny to provide even greater explanatory power. The methods proposed here can be applied to other organisms, at different hierarchical levels (family, infraspecific [110], [111]), sample regions and also for other properties, such as the search for new food plants [112], plants with economical potential [15], or new chemical compounds for medicine or pesticides [25], [26], [27], [113]. Future analyses can include ecological data that can predict in a phylogenetic context which areas harbour medicinal species diversity (medicinal hotspots). Phytochemical and ethnomedicinal data can be combined on phylogenies to test how well they can provide reciprocal illumination. Furthermore, similar studies can further our understanding of cultural processes that shape ethnobotanical knowledge, as phylogenetic similarity can be added as an extra parameter in cross-cultural comparisons of ethnomedicinal systems in order to provide greater insight into usage in different cultures.

Although ethnobotanically directed screening was proposed as a promising way of enhancing rates of bioprospecting schemes and several studies have shown that can lead to more positive hits compared to random sampling [3], [114], there are several reasons why these approaches are not likely to lead directly to new pharmaceutical drugs [115]. However, our study can serve as an example of how understanding patterns of successful traditional medicine can help promote local economic development through trade [116] appreciation of traditional medicine by the scientific community [117] and, most importantly, enhance local community health [118]. We would like to conclude with a reflection upon the ethical questions that arise where phylogenetic ethnobotany results in recovering successful traditional medicines. International legal frameworks, such as the one established by the Convention of Biological Diversity, safeguard the intellectual property of cultures and individuals with specialist knowledge. Profitable results from any such investigations should not only be profitable for both parts (investigators and people with knowledge), but must also focus on alleviating those people's livelihoods and enhance their healthcare [119]. A mechanism of benefit sharing is needed for cases where new medicinal plant discoveries that are not traditionally used in some culture but are based on traditional knowledge of species that are closely related to them.

## Supporting Information

Table S1 **Medicinal uses and properties of** ***Pterocarpus*** **species from the literature.**
(DOC)

Table S2 **Voucher specimen information of** ***Pterocarpus*** **samples and GenBank accession numbers.**
(XLS)

Table S3 **GenBank accession numbers for** ***Pterocarpus*** **outgroup samples.**
(XLS)

Table S4 **Matrix used for Phylocom analyses, scoring** ***Pterocarpus*** **taxa used per category of use.**
(XLS)
