# Supplementary material for: The Use of Phylogeny to Interpret Cross-Cultural Patterns in Plant Use and Guide Medicinal Plant Discovery: An Example from Pterocarpus (Leguminosae)
Source: PLoS One. 2011 Jul 18;6(7):e22275. doi: 10.1371/journal.pone.0022275 (PMC3138776; doi:10.1371/journal.pone.0022275)
Supplement: Table S1 — Medicinal uses and properties of Pterocarpus species from the literature. (DOC) [file pone.0022275.s001.doc]

**Table S1. Medicinal uses and properties of *Pterocarpus*** species from the literature.

| **Taxon** | **Uses** |
| --- | --- |
| ***P. acapulcensis*** | **Circulatory/ Blood** (diabetes [1]); **Gastro-intestinal** (astringent, gastric ulcers, haemorrhoids [1]); **Respiratory** (sore throat [2], tonsillitis [1]); **Skin** (sores [3]) |
| ***P. amazonum*** | **Infections/ Fevers** (fever [4], malaria *in vitro* [5]), **Skin** (burns [6], leishmaniasis [7], ulcers [8]) |
| ***P. angolensis*** | **Circulatory/ Blood** (anaemia [9]); **Gastro-intestinal** (abdominal pain [9], amoebic dysentery [9] *in vitro* [10] anthelmintic [9] *in vitro* [11], astringent [12], bloody diarrhoea [9], diarrhoea [12], dysentery [9]); **Genito-urinary/ Fertility** (abortifacient [9,13], amenorrhoea [9,14], female sterility [9], gonorrhoea [9,12], heavy menstruation [12], haematuria [9], hypermenorrhoea [9]); **Infections/ Fevers** (blackwater fever [9,12,15,16], malaria [9,12,15,16], schistosomiasis [9,12] *in vitro* [17]); **Inflammation** (anti-inflammatory [18], skin inflammation [9,16]); back pain [9], depressed fontanelle [9]); **Pain** (general body pain [9], headache [9,12]); **Respiratory** (asthma [9], cough [9]); **Sensory** (cataract [16], conjunctivitis [9]); **Skin** (head wounds [9,19], nettle rash [9], psoriasis [12,19], ringworm [9,20], skin problems [12], sores [9,12], ulcers [9,20], wounds [9,12,19]); **Unspeciﬁc** (antibacterial *in vitro* [10,11], galactagogue [9], malaise [21], unthriftiness [21]) |
| ***P. dalbergioides*** | **Circulatory/ Blood** (antidiabetic *in vitro* [22]) |
| ***P. erinaceus*** | **Circulatory/ Blood** (anaemia [9,12,23,24], blood tonic [25], haemostatic *in vitro* [26], high blood pressure [27]); **Gastro-intestinal** (amoebiasis [28], anthelmintic [12] *in vitro* [29], antiemetic [12], astringent for severe diarrhoea or dysentery [26], diarrhoea [12,30,31,32], dysentery [9,12,23,30,31,32,33], mouth diseases [9], purgative [9,12]); **Genito-urinary/ Fertility** (abortifacient [9], amenorrhoea [9], contraceptive [12] *in vitro* [34,35], dysmenorrhoea [9,23], difficult labour [9], female sterility [9], gonorrhoea [12,24], menstruation complaints [12,25], post partum haemorrhage [9,12], syphilis [12], urethral discharges [9], venereal diseases [9,12]); **Infections/ Fevers** (fever [9,12,31,36], malaria [9,12,37] *in vitro* [38]); **Inflammation** (anti-inflammatory *in vitro* [39]), **Musculo– skeletal** (body swelling [27], muscle relaxation *in vitro* [40]); **Nervous** (Alzheimer potential *in vitro* [23], fatigue [9], insomnia [31], learning retardation [23]); **Pain** (analgesic *in vitro* [39], headache [27], toothache [9,12]); **Poisons treatment** (antidote [27]); **Respiratory** (breathing improver [23], bronchial infections [12], cough [9], nose bleeding [27], sore throat [9], tuberculosis [12] *in vitro* [41]); **Sensory** (eye complaints [12]); **Skin** (bleeding wounds [26], chancre [9], ringworm infections [42], chronic ulcers [42], dermatomycosis [31], leprosy [12], ringworm infections [12] skin diseases [27], sores [12], ulcers [9,12], wound healing *in vitro* [12]); **Unspeciﬁc** (antibacterial *in vitro* [12,43], antioxidant *in vitro* [12,44], aphrodisiac [9,12], breast cancer [31], debility [9], insect repellent [12], panacea [9], restorative [9], tonic [12], tumours [12]) |
| ***P. indicus*** | **Circulatory/ Blood** (anaemia [45], blood circulation [46]); **Gastro-intestinal** (amoebic dysentery [47], anthelmintic [9], astringent [48], diarrhoea [48,49], dysentery [45,48], mouth sores [48,50], purgative [48]); bladder ailments [49], diuretic [48], kidney troubles [9], syphilitic sores [51]); **Infections/ Fevers** (malaria [48], single worm infection [9]); **Nervous** (exhaustion [47], neuralgia [47]); migraine/headache [9]); **Respiratory** (asthma [52], sore throat [48]); **Skin** (prickly heat [53], boils [53], dropsy [49], filariasis [54], male alopecia [55], sores [48], ulcers [53], wounds [48,50]); **Unspeciﬁc** (antibacterial *in vitro* [56], antifungal in vitro [57], cancers, especially of the mouth [58], tumours [58]) |
| ***P. lucens*** | **Gastro-intestinal** (diarrhoea [12], tapeworm [12]); **Genito-urinary/ Fertility** (kidney complaints [12]); **Musculo– skeletal** (lumbago [12]); **Pain** (headache [12]) |
| ***P. macrocarpus*** | **Infections/ Fevers** (malaria [59]) |
| ***P. marsupium*** | **Circulatory/ Blood** (diabetes [60,61] *in vitro* [62,63,64], cardiotonic activity *in vitro* [65]); **Gastro-intestinal** (bloody dysentery [66], diarrhoea [67], gastritis [61], stomach ache [68,69,70]); **Genito-urinary/ Fertility** (burning during urination [71], diuretic [72], fever with urinary tract infections [67], raise uterus [61]); joints pain [73], rheumatism [74]); **Nervous** (neurological problems [75]); **Pain** (toothache [67,76]); **Respiratory** (asthma [61], demulcent [72]); **Sensory** (cataract *in vitro* [77]); **Skin** (cracked lips [69], eczema [61], skin infections [67], wound healing [78]); **Unspeciﬁc** (anticancer activity *in vitro* [79]) |
| ***P. mildbraedii*** | **Unspeciﬁc** (antiageing [54]). |
| ***P. officinalis* subsp. *officinalis*** | **Circulatory/ Blood** (haemostatic [80]); **Gastro-intestinal** (astringent [7]); **Genito-urinary/ Fertility** (antifertility [81]); **Unspeciﬁc** (disinfectant [82]) |
| ***P. osun*** | **Circulatory/ Blood** (cholesterol *in vitro* [83], sickle-cell anaemia [12] *in vitro* [84,85]); **Genito-urinary/ Fertility** (amenorrhoea [9,12], candidiasis [31,54]); **Infections/ Fevers** (fever [31]); **Musculo– skeletal** (fractured bones [12,86], rheumatic complaints [12], sprains [12], stiff joints [12,86]); **Respiratory** (asthma [31]); **Skin** (acne [54], dermatomycosis [31], eczema [54], infections of umbilical cord [12,86], skin damage [87], skin diseases [12,30,86]); **Unspeciﬁc** (antiageing [54], antimicrobial activity *in vitro* [12,86], antioxidant [12]) |
| ***P. rohrii*** | **Infections/ Fevers** (fever [4], malaria [88], *in vitro* [89]); **Respiratory** (coughs [90]); **Skin** (wounds [91], ulcers [90]); **Unspeciﬁc** (antimicrobial activity *in vitro* [92]) |
| ***P. rotundifolius* subsp. *polyanthus*** | **Circulatory/ Blood** (anaemia [9]) |
| ***P. rotundifolius* subsp. r*otundifolius*** | **Genito-urinary/ Fertility** (impotence [54]); **Sensory** (eye problems [9]) |
| ***P. santalinoides*** | **Circulatory/ Blood** (sickle-cell anaemia [93]); **Gastro-intestinal** (amoebic dysentery [9,12], astringent [31,94], haemorrhoids [9,12], meterorism [9], stomach ache [12]); **Genito-urinary/ Fertility** (childbirth [12], circumcision [9], dystocia [9], infertility [94], threatened abortion [9,12]); **Infections/ Fevers** (fever [9,12,31,94,95], malaria [12] *in vitro* [96]); **Nervous** (nervous sedative [94], tiredness [33]); **Poisons treatment** (poison antidote [97], snake bite [9]); **Respiratory** (bronchial infections [12]); **Skin** (skin diseases [31], wounds [9,12]); **Unspeciﬁc** (asthenia [9], tonic [12]) |
| ***P. santalinus*** | **Circulatory/ Blood** (blood diseases [98,99] *in vitro* [100], blood purifier [101], diabetes [72,102] *in vitro* [100,103,104]); **Gastro-intestinal** (anticholinergic agent *in vitro* [105], astringent [99,106,107], bilious affections [99,107], bleeding haemorrhoids [72], bowel disorder [108], chronic dysentery [99,107], diarrhoea [107], dysentery [72], hepatoprotective *in vitro* [109,110], indigestion [108], haemorrhoids [111], ulcers *in vitro* [112], vomiting [98,99]); **Genito-urinary/ Fertility** (menstrual disorders [108]); **Infections/ Fevers (**cooling [107], diaphoretic [99,107]); **Inflammation** (anti-inflammatory *in vitro* [113], skin inflammations [72]); **Musculo– skeletal** (stiff neck [108]); **Pain** (headache [99,106,108]); **Poisons treatment** (antidote [98,99,101], scorpion sting [99,106,114], snake bites [114]); **Respiratory** (colds [108], coughs [108]); **Sensory** (eye diseases [98,99], sight improvement [99,106]); **Skin** (blisters [115], boils [99,115], eczema [108], rashes [108], skin diseases [99,101,107], wound healing [78,99] *in vitro* [116]); **Unspeciﬁc** (antioxidant *in vitro* [117], aphrodisiac [98,99], tonic [98,99,106,107]) |
| ***P. soyauxii*** | **Circulatory/ Blood** (anaemia [118], diabetes [119], hypertension [119]); **Gastro-intestinal** (anorexia [9], diarrhoea [9], dysentery [9,12], gastralgia [118], haemorrhoids [9,12], hernia [9,12], intestinal parasites [9,119], lumbar pains [120]); **Genito-urinary/ Fertility** (bloody blenorrhagia [9], childbirth [121], diuretic [119], dysmenorrhoea [9,12], gonorrhea [122], hypermenorrhoea [9,12], uterine haemorrhage [9,12]); **Inflammation** (anti-inflammatory [12,54]); **Musculo– skeletal** (rheumatism [123]); **Nervous** (stimulant [9]); **Pain** (toothache [9,12]); **Respiratory** (bronchitis [9], broncho-pulmonary problems [9,12], measles [9], renal diseases [119]); **Skin** (cicatrisation [9], cutaneous diseases [119], favus [9], oedemas [9,12], scabies [9], whitlow [9,12], wounds [9], yaws [9]); **Unspeciﬁc** (antifungal activity [12], degenerative diseases [9], tonic in jaundice [124]) |
| ***P. tinctorius*** | **Gastro-intestinal** (dysentery [9], haemorrhoids [9], hernia [9]); **Genito-urinary/ Fertility** (dysmenorrhoea [9], uterine haemorrhage [9]); **Respiratory** (bronchitis [9], pulmonary complaints [9], pulmonary congestion [9,12], respiratory diseases [125]); **Sensory** (conjunctivitis [9]); **Skin** (oedemas [9], whitlow [9]). |

# References S1

1. Giraldo D, Baquero E, Bermúdez A, Oliveira-Miranda MA (2009) Medicinal plant trade characterization in popular markets of Caracas, Venezuela. Acta Botanica Venezuelica 32: 267-301.

2. Bermúdez A, Velázquez D (2002) Etnobotánica médica de una comunidad campesina del estado Trujillo, Venezuela: un estudio preliminar usando técnicas cuantitativas. Revista de la Facultad de Farmacia 44: 2-6.

3. Diaz P. WA (2007) Preliminary inventory of useful plants of remain forests from Las Delicias and El Guamo, Serranía Imataca, Bolívar State, Venezuela. Acta Botanica Venezuelica 30: 327-344.

4. Milliken W (1997) Plants for malaria, plants for fever. London: Royal Botanic Gardens Kew.

5. Muñoz V, Sauvain M, Bourdy G, Callapa J, Bergeron S, et al. (2000) A search for natural bioactive compounds in Bolivia through a multidisciplinary approach: Part I. Evaluation of the antimalarial activity of plants used by the Chacobo Indians. Journal of Ethnopharmacology 69: 127-137.

6. Bergeron S, Ortiz J, Ortiz B, Soria C (1997) El uso de las plantas por los Chacobos (Alto Ivon, Beni, Bolivia). La Paz, Bolivia: Editions IBIS Dinamarca.

7. Pieters L (1998) La "sangre de drago", una droga tradicional de Sudamerica. Constituyentes biologicamente activos. Quito, Ecuado: Ediciones Abya-Yala.

8. Fenner R, Heemann Betti A, Auler Mentz L, Maris Kuze Rates S (2006) Plants with potencial antifungal activity employed in Brazilian folk medicine. Brazilian Journal of Pharmaceutical Sciences 42: 369-394.

9. Neuwinger HD (2000) African traditional medicine. Stuttgart, Germany: Medpharm Scientific Publishers.

10. Ramalivhana JN, Moyo SR, Obi CL (2010) The possible role of medicinal plants in tackling resistant microbial pathogens in Limpopo Province, South Africa. Journal of Medicinal Plants Research 4: 999-1002.

11. McGaw LJ, Van der Merwe D, Eloff JN (2007) *In vitro* anthelmintic, antibacterial and cytotoxic effects of extracts from plants used in South African ethnoveterinary medicine. The Veterinary Journal 173: 366-372.

12. Gazzaneo L, de Lucena R, de Albuquerque U (2005) Knowledge and use of medicinal plants by local specialists in an region of Atlantic Forest in the state of Pernambuco (Northeastern Brazil). Journal of Ethnobiology and Ethnomedicine 1: 9.

13. Bally PRO (1937) Native medicinal and poisonous plants of East Africa. Bulletin of Miscellaneous Information Royal Botanical Garden 1: 10-26.

14. Steenkamp V (2003) Traditional herbal remedies used by South African women for gynaecological complaints. Journal of Ethnopharmacology 86: 97-108.

15. Palgrave KC (1957) The trees of Central Africa. Salisbury: National Publications Trust, Rhodesia and Nyasaland.

16. Watt JM, Breyer-Brandwijk MG (1962) The medicinal and poisonous plants of South and East Africa. Edinburgh and London: E. and S. Livingstone.

17. Ndamba J, Nyazema N, Makaza N, Anderson C, Kaondera KC (1994) Traditional herbal remedies used for the treatment of urinary schistosomiasis in Zimbabwe. Journal of Ethnopharmacology 42: 125-132.

18. Recio MC, Giner RM, Mánez S, Rios JL, Marston A, et al. (1995) Screening of tropical medicinal plants for antiinflammatory activity. Phytotherapy Research 9: 571-574.

19. Hutchings A, Scott AH, Lewis G, Cunningham A (1996) Zulu medicinal plants: An inventory. Pietermaritzburg: University of Natal Press.

20. Van der Reit K, van Rensburg L, De Sousa Correia RI, L.J. M, Kruger GHJ (1998) Germination of *Pterocarpus angolensis* DC. and evaluation of the possible antimicrobial action of the phloem sap. South African Journal of Plant and Soil 15: 141-146.

21. Luseba D, Van der Merwe D (2006) Ethnoveterinary medicine practices among Tsonga speaking people of South Africa. Onderstepoort Hournal of Veterinary Research 73: 115-122.

22. Murthy YLN, Viswanadh GS, Atchuta Ramaiah P, Chandra Sekhar Naidu K (2004) Antidiabetic activity of heartwood extract of *Pterocarpus dalbergioides*. Journal of Tropical Medicinal Plants 4.

23. Hage S, Kienlen-Campard P, Octave J-N, Quetin-Leclercq J (2010) In vitro screening on β-amyloid peptide production of plants used in traditional medicine for cognitive disorders. Journal of Ethnopharmacology 131: 585-591.

24. Abreu PM, Martins ES, Kayser O, K.-U. B, Siems K, et al. (1999) Antimicrobial, antitumor and antileishmania screening of medicinal plants from Guinea-Bissau. Phytomedicine 6.: 187-195.

25. Amusa TO, Jimoh SO, Aridanzi P, Haruna M (2010) Ethnobotany and conservation of plant resources of Kainji Lake National Park, Nigeria. Ethnobotany Research & Applications 8: 181-194.

26. Salawu O, Aliyu M, Tijani A (2008) Haematological studies on the ethanolic stem bark extract of *Pterocarpus erinaceus* Poir. (Fabaceae). African Journal of Biotechnology 7: 1212-1215.

27. Nadembega P, Boussim JI, Nikiema JB, Poli F, Antognoni F (2011) Medicinal plants in Baskoure, Kourittenga Province, Burkina Faso: An ethnobotanical study. Journal of Ethnopharmacology 133: 378-395.

28. Jiofack T, Fokunang C, Guedje N, Kemeuze V, Fongnzossie E, et al. (2010) Ethnobotanical uses of medicinal plants of two ethnoecological regions of Cameroon. International Journal of Medicine and Medical Sciences 2: 60-79.

29. Waterman C, Smith RA, Pontiggia L, DerMarderosian A (2010) Anthelmintic screening of Sub-Saharan African plants used in traditional medicine. Journal of Ethnopharmacology 127: 755-759.

30. Kayode J (2006) Conservation of indigenous medicinal botanicals in Ekiti State, Nigeria. Journal of Zhejiang University SCIENCE B 7: 713-718.

31. Olowokudejo JD, B. KA, Travih VA (2008) An ethnobotanical survey of herbal markets and medicinal plants in Lagos state of Nigeria. Ethnobotanical Leaflets 12: 851-865.

32. Chinsembu KC, Hedimbi M (2010) An ethnobotanical survey of plants used to manage HIV/AIDS opportunistic infections in Katima Mulilo, Caprivi region, Namibia. Journal of Ethnobiology and Ethnomedicine 6.

33. Allabi AC, Busia K, Ekanmian V, Bakiono F (2011) The use of medicinal plants in self-care in the Agonlin region of Benin. Journal of Ethnopharmacology 133: 234-243.

34. Benie T, Thieulant ML (2003) Interaction of some traditional plant extracts with uterine oestrogen or progestin receptors. Phytotherapy Research 17: 756-760.

35. Benie T, Duval J, Thieulant ML (2003) Effects of some traditional plant extracts on rat oestrous cycle compared with Clomid. Phytotherapy Research 17: 748-755.

36. Irvine FR (1961) Woody plants of Ghana. London Oxford University Press.

37. Asase A, Oteng-Yeboah AA, Odamtten GT, Simmonds MSJ (2005) Ethnobotanical study of some Ghanaian anti-malarial plants. Journal of Ethnopharmacology 99: 273-279.

38. Karou D, Dicko MH, Sanon S, Simpore J, Traore AS (2003) Antimalarial activity of *Sida acuta* Burm. f. (Malvaceae) and *Pterocarpus erinaceus* Poir. (Fabaceae). Journal of Ethnopharmacology 89: 291-294.

39. Aliyu M, Salawu O, Wannang N, Yaro A, Bichi L (2005) Analgesic and anti-inflammatory activities of the ethanolic extract of the stem bark of *Pterocarpus erinaceus* in mice and rats. Nigerian Journal of Pharmaceutical Research 4: 12-17.

40. Aliyu M, Chedi B (2010) Effects of the ethanolic stem bark extract of *Pterocarpus erinaceus* Poir. (Fabaceae) on some isolated smooth muscles. Bayero Journal of Pure and Applied Sciences 3: 34-38.

41. Uba A, Ibrahim K, Agbo EB, Makinde AA (2003) In vitro inhibition of *Mycobacterium smegmatis* and *Mycobacterium tuberculosis* by some Nigerian medicinal plants. East and Central African Journal of Pharmaceutical Sciences 6: 15-19.

42. Dalziel J (1948) The Useful Plants of West Tropical Africa. London: The Crown Agents for the colonies.

43. Dramane S, Mamidou Witabouna K, Kagoyire K (2010) Evaluation of antimicrobial and free radical scavenging activities of some bioactive taxa from Côte D’ivoire. European Journal of Scientific Research 40: 307-317.

44. Karou D, Dicko MH, Simpore J, Traore AS (2005) Antioxidant and antibacterial activities of polyphenols from ethnomedicinal plants of Burkina Faso. African Journal of Biotechnology 4: 823-828.

45. Timi D (1994) Medicinal plant survey of Papua New Guinea. In: Morrison J, Geraghty PA, Crow l, editors. Science of Pacific island poples: Fauna, flora, food and medicine. Suva, Fiji: Institute of Pacific Studies. pp. 185-200.

46. Delang CO (2007) The role of medicinal plants in the provision of healthcare in Lao PDR. Journal of Medicinal Plants Research 1: 50-59.

47. Defilipps RA, Maina SL, Pray LA (1988) The Palauan and Yap medicinal plant studies of Masayoshi Okabe 1941-1943. Washington D.C., USA: National Museum of Natural History, Smithsonian Institution.

48. Perry L (1980) Medicinal plants of East and Southeast Asia: attributed properties and uses. Cambridge, Massachusetts, and London: The MIT Press.

49. Duke J, Wain K (1981) Medicinal plants of the world; Computer Index with more than 85,000 entries. London, UK: Longman group UK Limited.

50. Collins S, Martins X, Mitchell A, Teshome A, Arnason J (2007) Fataluku medicinal ethnobotany and the East Timorese military resistance. Journal of Ethnobiology and Ethnomedicine 3: 5.

51. Burkill JH (1966) A dictionary of economic products of the Malay Peninsula. Kuala Lumpur: Art Printing Works.

52. Ecarma VV (2004) Method to enhance the immune system and used for the prevention and treatment of asthma. Patent in the U.S.A.

53. Carandang WM *Pterocarpus indicus* Willd. Kuala Lumpur: APFORGEN Secretariat.

54. Lawal IO, Uzokwe NE, Igboanugo ABI, Adio AF, E. A. Awosan1, et al. (2010) Ethno medicinal information on collation and identification of some medicinal plants in Research Institutes of South-west Nigeria. African Journal of Pharmacy and Pharmacology 4: 1-7.

55. Dang J, Yu K, Wang Y (2010) Study on treatment of *Pterocarpus indicus* Willd. extracts on rat androgenetic alopecia. West China Journal of Pharmaceutical Sciences 3.

56. Khan MR, Omoloso AD (2003) Antibacterial activity of *Pterocarpus indicus*. Fitoterapia 74: 603-605.

57. Kawamura F, Mahamud A, Sulaiman O, Hashim R (2010) Antifungal activities of extracts from heartwood, sapwood and bark of 11 Malaysian timbers against *Gleophyllum trabeum* and *Pycnoporus sanguineus*. Journal of Tropical Forest Science 22: 170-174.

58. Hartwell JL (1967-1971) Plants used against cancer. A survey. Lloydia: 30-34.

59. Nguyen-Pouplin J, Tran H, Tran H, Phan TA, Dolecek C, et al. (2007) Antimalarial and cytotoxic activities of ethnopharmacologically selected medicinal plants from South Vietnam. Journal of Ethnopharmacology 109: 417-427.

60. Grover JK, Yadav S, Vats V (2002) Medicinal plants of India with anti-diabetic potential. Journal of Ethnopharmacology 81: 81-100.

61. Jain A, Katewa SS, Galav PK, Sharma P (2005) Medicinal plant diversity of Sitamata wildlife sanctuary, Rajasthan, India. Journal of Ethnopharmacology 102: 143-157.

62. Vats V, Grover JK, Rathi SS (2002) Evaluation of anti-hyperglycemic and hypoglycemic effect of *Trigonella foenum-graecum* Linn, *Ocimum sanctum* Linn and *Pterocarpus marsupium* Linn in normal and alloxanized diabetic rats. Journal of Ethnopharmacology 79: 95-100.

63. Kar A, Choudhary BK, Bandyopadhyay NG (2003) Comparative evaluation of hypoglycaemic activity of some Indian medicinal plants in alloxan diabetic rats. Journal of Ethnopharmacology 84: 105-108.

64. Dhanabal SP, Kokate CK, Ramanathan M, Kumar EP, Suresh B (2006) Hypoglycaemic activity of *Pterocarpus marsupium* Roxb. Phytotherapy Research 20: 4-8.

65. Mohire NC, Salunkhe VR, Bhise SB, Yadav AV (2007) Cardiotonic activity of aqueous extract of heartwood of *Pterocarpus marsupium*. Indian Journal of Experimental Biology 45: 532-537.

66. Sen SK, Behera LM (2008) Ethnomedicinal plants used by the tribals of Bargarh district to cure diarrhoea and dysentery. Indian Journal of Traditional Knowledge 7: 425-428.

67. Jain SK (1968) Medicinal Plants. New Delhi, India: National Book Trust.

68. Singh AK, Raghubanshi AS, Singh JS (2002) Medical ethnobotany of the tribals of Sonaghati of Sonbhadra district, Uttar Pradesh, India. Journal of Ethnopharmacology 81: 31-41.

69. Nayak S, Behera SK, Misra MK (2004) Ethno-medico-botanical survey of Kalahandi district of Orissa. Indian Journal of Traditional Knowledge 3: 72-79.

70. Rout SD, Panda T, Mishra N (2009) Ethno-medicinal plants used to cure different diseases by tribals of Mayurbhanj District of North Orissa. Studies on Ethno-Medicine 3: 27-32

71. Punjani BL (2010) Herbal folk medicines used for urinary complaints in tribal pockets of Northeast Gujarat. Indian Journal of Traditional Knowledge 9: 126-130.

72. Nadkarni AK, Nadkarni KM (1976) Indian materia medica. Bombay: Popular Prakashan.

73. Sutha S, Mohan VR, Kumaresan S, Murugan C, Athiperumalsami T (2010) Ethnomedicinal plants used by the tribals of Kalakad-Mundanthurai Tiger Reserve (KTMR), Western Ghats, Tamil Nadu for the treatment of rheumatism. Indian Journal of Traditional Knowledge 9: 502-509.

74. Udayan PS, Tushar KV, George S, Balachandran I (2007) Ethnomedicinal information from Kattunayakas tribes of Mudumalai Wildlife Sanctuary, Nilgiris district, Tamil Nadu. Indian Journal of Traditional Knowledge 6: 574-578.

75. Acharya KP, Rokaya MB (2005) Ethnobotanical survey of medicinal plants traded in the streets of Kathmandu valley. Scientific World 3: 44-48.

76. Ganesan S (2008) Traditional oral care medicinal plants survey of Tamil Nadu. Indian Journal of Natural Products and Resources 7: 166-172.

77. Vats V, Yadav SP, Biswas NR, Grover JK (2004) Anti-cataract activity of *Pterocarpus marsupium* bark and *Trigonella foenum-graecum* seeds extract in alloxan diabetic rats. Journal of Ethnopharmacology 93: 289-294.

78. Chopda MZ, Mahajan RT (2009) Wound healing plants of Jalgaon District of Maharashtra State, India. Ethnobotanical Leaflets 13: 1-32.

79. Chakraborty A, Gupta N, Ghosh K, Roy P (2010) In vitro evaluation of the cytotoxic, anti-proliferative and anti-oxidant properties of pterostilbene isolated from *Pterocarpus marsupium*. Toxicology in Vitro 24: 1215-1228.

80. Little EL (1964) Common trees of Puerto Rico and the Virgin Islands. Washington D.C., USA: Department of Agriculture.

81. Weniger B, Haag-Berrurier M, Anton R (1982) Plants of Haiti used as antifertility agents. Journal of Ethnopharmacology 6: 67-84.

82. Lioger AH (1990) Plantas medicinales de Puerto Rico y del Caribe. San Juan: Iberoambericana de Ediciones, Inc.

83. Rimando AM, Nagmani R, Feller DR, Yokoyama W (2005) Pterostilbene, a new agonist for the peroxisome proliferator-activated receptor α-isoform, lowers plasma lipoproteins and cholesterol in hypercholesterolemic hamsters. Journal of Agricultural and Food Chemistry 53: 3403-3407.

84. Obodozie OO, Ameh SJ, Afolabi EK, Oyedele EO, Ache TA, et al. (2010) A normative study of the components of Niprisan—an herbal medicine for sickle cell anemia. Journal of Dietary Supplements 7: 21-30.

85. Nathan S, Tripathi P, Wu Q, Belanger Faith C (2009) Nicosan: Phytomedicinal treatment for sickle cell disease. African Natural Plant Products: New Discoveries and Challenges in Chemistry and Quality: American Chemical Society. pp. 263-276.

86. Ebi GC, Ofoefule SI (2000) Antimicrobial activity of *Pterocarpus osun* stems. Fitoterapia 71: 433-435.

87. Olukemi O, Oluseyi JM, Olukemi IO, Olutoyin SM (2005) The use of selected Nigerian natural products in the management of environmentally Induced free radicals skin damage. Pakistan Journal of Biological Sciences 8: 1074-1077.

88. Schultes RE, Raffauf RF (1990) The healing forest: Medicinal and toxic plants of the Northwest Amazonia. Portland: Dioscorides Press.

89. Bertani S, Bourdy G, Landau I, Robinson JC, Esterre P, et al. (2005) Evaluation of French Guiana traditional antimalarial remedies. Journal of Ethnopharmacology 98: 45-54.

90. de Fátima Agra M, de Freitas PF, Barbosa-Filho JM (2007) Synopsis of the plants known as medicinal and poisonous in Northeast of Brazil. Brazilian Journal of Pharmacognosy 17: 114-140.

91. Reynel C, Pennington TD, Pennington RT, Flores C, Daza A (2003) Arboles utiles de la Amazonia Peruana y sus usos. Lima, Peru: Tarea Grafica Educativa.

92. Kloucek P, Svobodova B, Polesny Z, Langrova I, Smrcek S, et al. (2007) Antimicrobial activity of some medicinal barks used in Peruvian Amazon. Journal of Ethnopharmacology 111: 427-429.

93. Okpuzor J, Adebesin O, Ogbunugafor H, Amadi I (2008) The potential of medicinal plants in sickle cell disease control: A review. International Journal of Biomedical and Health Sciences 4: 47-55.

94. Lawal IO, Igboanugo ABI, Osikarbor B, Duyilemi OP, Adesoga AA, et al. (2010) Evaluation of plant-based non-timber forest products (ntfps) as potential bioactive drugs in South-western Nigeria. Journal of Clinical Medicine and Research 3: 61-66.

95. Bouquet A, Debray M (1974) Plantes medicinales de la Cote d'lvoire. Paris: O.R.S.T.O.M.

96. Valentin A, Mustofa, Benoit-Vical F, Pélissier Y, Koné-Bamba D, et al. (2000) Antiplasmodial activity of plant extracts used in west African traditional medicine. Journal of Ethnopharmacology 73: 145-151.

97. Rodrigues E, Mendes FR, Negri G (2006) Plants indicated by Brazilian Indians to Central Nervous System disturbances: A bibliographical approach. Current Medicinal Chemistry - Central Nervous System Agents 6: 211-244.

98. Kirtikar KR, Basu BD (2001) Indian medicinal plants - With Illustrations. Second edition. Dehradun, India: Oriental Enterprises.

99. Chopra RN, Nayar SL, Chopra IC (1956) Glossary of Indian medicinal plants. New Delhi, India: CSIR Publications.

100. Kondeti VK, Badri KR, Maddirala DR, Thur SKM, Fatima SS, et al. (2010) Effect of *Pterocarpus santalinus* bark, on blood glucose, serum lipids, plasma insulin and hepatic carbohydrate metabolic enzymes in streptozotocin-induced diabetic rats. Food and Chemical Toxicology 48: 1281-1287.

101. Yesodharan K, Sujana KA (2007) Ethnomedicinal knowledge among Malamalasar tribe of Parambikulam wildlife sanctuary, Kerala. Indian Journal of Traditional Knowledge 6: 481-485.

102. Nagaraju N, Rao KN (1990) A survey of plant crude drugs of Rayalaseema, Andhra Pradesh, India. Journal of Ethnopharmacology 29: 137-158.

103. Kameswara Rao B, Giri R, Kesavulu MM, Apparao C (2001) Effect of oral administration of bark extracts of *Pterocarpus santalinus* L. on blood glucose level in experimental animals. Journal of Ethnopharmacology 74: 69-74.

104. Nagaraju N, Prasad M, Gopalakrishna G, Rao KN (1991) Blood sugar lowering effect of *Pterocarpus santalinus* (Red Sanders) wood extract in different rat models. Pharmaceutical Biology 29: 141-144.

105. Narayan S, Devi RS, Ganapathi V, Devi CSS (2007) Effect of *Pterocarpus santalinus* extract on the gastric pathology elicited by a hypertensive drug in wistar rats. Pharmaceutical Biology 45: 468-474.

106. Latheef SA, Prasad, B., Bavaji, M., Subramanyam, G. (2008) A database on endemic plants at Tirumala hills in India. Bioinformation 2: 260-262.

107. Krishna Murthi A (1969) The Wealth of India. New Delhi, India: CSIR.

108. Awale S, Linn TZ, Than MN, Swe T, Saiki I, et al. (2006) The healing art of traditional medicines in Myanmar. Journal of Traditional Medicine: 47-68.

109. Palanisamy D, Syamala, Kannan E, Bhojraj S (2007) Protective and therapeutic effects of the Indian medicinal plant *Pterocarpus santalinus* on D-galactosamine-induced liver damage. Asian Journal of Traditional Medicines 2: 51-57.

110. Manjunatha BK (2006) Hepatoprotective activity of *Pterocarpus santalinus* L.f., an endangerd medicinal plant. Indian Journal of Pharmacology 38: 25-28.

111. Behera SK, Panda A, Behera SK, Misra MK (2006) Medicinal plants used by the Kandhas of Kandhamal district of Orissa. Indian Journal of Traditional Knowledge 5: 519-528.

112. Narayan S, Devi RS, Srinivasan P, Devi CSS (2005) *Pterocarpus santalinus*: a traditional herbal drug as a protectant against ibuprofen induced gastric ulcers. Phytotherapy Research 19: 958-962.

113. Cho JY, PARK J, Su KIM P, YOO ES, BAIK KU, et al. (2001) Savinin, a lignan from *Pterocarpus santalinus* inhibits tumor necrosis factor-α production and T cell proliferation. Biological & Pharmaceutical Bulletin 24: 167-171.

114. Warrier PK, Nambiar VPK, Raman Kutty C (1994) Indian Medicinal Plants Vol 2. Hyderabad: Orient Longman Limited.

115. Rao DM, Rao UVUB, Sudharshanam G (2006) Ethno-medico-botanical studies from Rayalaseema region of southern Eastern Ghats, Andhra Pradesh, India. Ethnobotanical Leaflets 10: 198-207.

116. Biswas TK, Maity LN, Mukherjee B (2004) Wound healing potential of *Pterocarpus santalinus* Linn: A pharmacological evaluation. The International Journal of Lower Extremity Wounds 3: 143-150.

117. Arokiyaraj S, Martin S, Perinbam K, Marie Arockianathan P, Beatrice V (2008) Free radical scavenging activity and HPTLC finger print of *Pterocarpus santalinus* L. - an *in vitro* study Indian Journal of Science and Technology 7: 1-3.

118. Jiofack T, Ayissi l, Fokunang C, Guedje N, Kemeuze V (2009) Ethnobotany and phytomedicine of the upper Nyong valley forest in Cameroon. African Journal of Pharmacy and Pharmacology 3: 144-150.

119. Tchamadeu MC, Dzeufiet PDD, Nana P, Kouambou Nouga CC, Ngueguim Tsofack F, et al. (2011) Acute and sub-chronic oral toxicity studies of an aqueous stem bark extract of *Pterocarpus soyauxii* Taub (Papilionaceae) in rodents. Journal of Ethnopharmacology 133: 329-335.

120. Nyakabwa M, Dibaluka M (1990) Plantes medicinales cultivees dans la zone de Kabondo a Kisangani (Zaire). African Study Monographs 12: 87-99.

121. Eyong CT (2007) Indigenous knowledge and sustainable development in Africa: Case study on central Africa. In: Boon EK, Hens L, editors. Indigenous knowledge systems and sustainable development: Relevance for Africa Tribes and tribals special volume: Kamla-Raj Enterprises. pp. 121-140.

122. Betti JL (2004) An ethnobotanical study of medicinal plants in the Baka Pygmies in the Dja Biosphere Reserve, Cameroon. African Study Monographs 25: 1-27.

123. Adjanohoun EJ, Ake Assi L, Chibon P, De Vecchy H, Goudote E, et al. (1985) Contribution aux études ethnobotaniques et floristiques au Gabon. (Médicine traditionelle et pharmacopée). Paris: A.C.C.T.

124. Betti JL, Lejoly J (2009) Contribution to the knowledge of medicinal plants of the Dja Biosphere Reserve, Cameroon: Plants used for treating jaundice. Journal of Medicinal Plants Research 3: 1056-1065.

125. Disengomoka I, Delaveau P, Sengele K (1983) Medicinal plants used for child's respiratory diseases in Zaire. Part II. Journal of Ethnopharmacology 8: 265-277.
